# A case of rapidly progressive IgA nephropathy in a patient with exacerbation of Crohn’s disease

**DOI:** 10.1186/1471-2369-13-84

**Published:** 2012-08-06

**Authors:** Ji-Young Choi, Chung Hoon Yu, Hee-Yeon Jung, Min Kyu Jung, Yong-Jin Kim, Jang-Hee Cho, Chan-Duck Kim, Yong-Lim Kim, Sun-Hee Park

**Affiliations:** 1 Division of Nephrology, Department of Internal Medicine, Kyungpook National University School of Medicine and Clinical Research Center for End Stage Renal Disease in Korea, 130 Dongduk-ro, Jung-gu, Daegu, South Korea; 2 Division of Gastroenterology, Department of Internal Medicine, Kyungpook National University School of Medicine, Daegu, South Korea; 3 Department of Pathology, Yeungnam University School of Medicine, Daegu, South Korea

**Keywords:** Rapidly progressive IgA nephropathy, Crohn’s disease

## Abstract

**Background:**

IgA nephropathy has been reported as a renal involvement in Crohn’s disease. Crescentic IgA nephropathy, which accounts for fewer than 5% of cases of IgA nephropathy, has a poorer prognosis than other forms of crescentic glomerulonephritis. We recently experienced a case of rapidly progressive IgA nephropathy concurrent with exacerbation of Crohn’s disease.

**Case presentation:**

An 18-year-old male diagnosed with Crohn’s disease underwent a hemicolectomy 2 years prior previously. He had maintained a state of Crohn’s disease remission with 5-aminosalicylic acid treatment. Four months prior to referral to the nephrology clinic, he experienced non-bloody diarrhea. He simultaneously developed proteinuria and microscopic hematuria with deterioration of renal function. Based on renal biopsy findings, the patient was diagnosed with crescentic IgA nephropathy. Immunostaining for interleukin-17 in renal tissue and previous exacerbated colonic ulcers was positive. Steroid pulse therapy was administered, followed by high-dose glucocorticoid and oral cyclophosphamide therapy. The patient’s renal function recovered and his gastrointestinal symptoms were alleviated.

**Conclusions:**

We report a case of crescentic IgA nephropathy presenting with exacerbation of Crohn’s disease, and present a review of the literature focusing on the pathophysiologic relationship between these two conditions.

## Background

Renal involvement, including tubulointerstitial nephritis, amyloidosis, and urolithiasis caused by calcium oxalate or urate, has been reported in approximately 4% to 23% of patients with Crohn’s disease [1,2]. IgA nephropathy (IgAN) is a renal disease associated with inflammatory bowel diseases such as Crohn’s disease. Several cases of IgAN accompanying Crohn’s disease have been reported [3-7] in which patients developed proteinuria and hematuria with or without mild deterioration of renal function.

IgAN, a slowly progressive glomerular disorder, can lead to end-stage renal disease within 20 years of diagnosis in up to 25% to 30% of patients with this condition. Although fewer than 5% of IgAN patients show crescentic or rapidly progressive IgAN [8,9], crescentic IgAN has a poorer prognosis than other forms of crescentic glomerulonephritis [9].

Crescentic IgAN has rarely been observed in patients with Crohn’s disease. Here, we report a patient with rapidly progressive IgAN and exacerbation of Crohn’s disease. His intestinal symptoms and renal functions both improved after the administration of immunosuppressive agents. To better understand the pathogenesis, we also review and summarize previous reports highlighting the pathophysiological relationship between these two conditions.

## Case presentation

An 18-year-old male was referred to our nephrology clinic for evaluation of azotemia and proteinuria in March 2011. The patient had previously undergone an operation to repair an anal fistula in December 2008 and an appendectomy to treat acute appendicitis in January 2009 at a local clinic. He was admitted to our hospital in June 2009 due to right upper quadrant pain and bloody diarrhea. Urinalysis revealed an absence of proteinuria and microscopic hematuria. There was no deterioration of renal function as determined by blood urea nitrogen (BUN, 5.9 mg/dL) and serum creatinine (0.9 mg/dL) analysis. Colonoscopic examination revealed linear, geographic ulcers with non-caseating granulomas in the distal ileum. The patient was diagnosed with Crohn’s disease and treated with 3 g/day of 5-aminosalicylic acid (5-ASA, mesalazine) and 9 mg/day of budesonide.

In July 2009, the patient visited the emergency room with right lower quadrant pain, loose stool, and fever. Simple abdominal X-ray showed free air below the diaphragm. He was diagnosed with an ascending colon perforation and underwent an emergency right hemicolectomy. Budesonide administration was discontinued, and the 5-ASA dosage was reduced to 2 g/day. The patient was additionally treated with ciprofloxacin (1 g/day) and metronidazole (1.5 g/day), and was kept under observation for 2 weeks. After this time, he had intermittent loose stools (0–2 times/day) without abdominal pain. Next, the patient was given 5-ASA (2 g/day) as maintenance therapy and his intestinal symptoms improved.

In December 2010, the patient experienced exacerbation of his symptoms, with non-bloody diarrhea three to four times daily. Colonoscopy revealed geographic ulcers consistent with Crohn’s disease. His blood pressure was 127/76 mmHg. Laboratory test results showed normal BUN (10.1 mg/dL) and serum creatinine (0.76 mg/dL) levels. Although urinalysis revealed mild proteinuria (1+ according to a dipstick test) and microscopic hematuria, the patient did not have a nephrology consultation. The patient was treated with 5-ASA (1 g/day) and budesonide (6 mg/day). After treatment, his abdominal pain ceased and no more loose stools were experienced until March 2011.

In March 2011, the patient experienced dark loose stools three times daily, and developed pale conjunctiva. He complained of facial puffiness and edema in his lower extremities. His blood pressure was 137/96 mmHg. Proteinuria (2+ by a dipstick test) and microscopic hematuria were detected by urinalysis. His renal function had deteriorated, with increased BUN (30.3 mg/dL) and creatinine (3.42 mg/dL) levels. He was referred to a nephrology clinic and admitted for evaluation.

His height was 170 cm and his body weight was 43 kg. A 24-h urine study revealed the presence of proteinuria (3,967 mg/day). Further serologic testing revealed a mildly increased IgA level of 492 mg/dL (reference, 70–400 mg/dL) along with normal IgG (670 mg/dL; reference, 700–1,600 mg/dL), IgM (55 mg/dL; reference, 40–230 mg/dL), C3 (126.7 mg/dL; reference, 90–180 mg/dL), and C4 (40.5 mg/dL; reference, 10–40 mg/dL) levels. Test results for anti-nuclear antibodies (ANA), anti-neutrophil cytoplasmic antibodies (ANCA), anti-glomerular basement membrane (anti-GBM) antibodies, anti-streptolysin O (ASO) antibodies, rheumatoid factor (RF), and cryoglobulin were all negative. Serologic tests for hepatitis B, hepatitis C, and human immunodeficiency viruses were also negative. Renal ultrasonographic findings showed that the kidneys were normal-sized, with increased cortical echogenicity.

**Figure 1:**
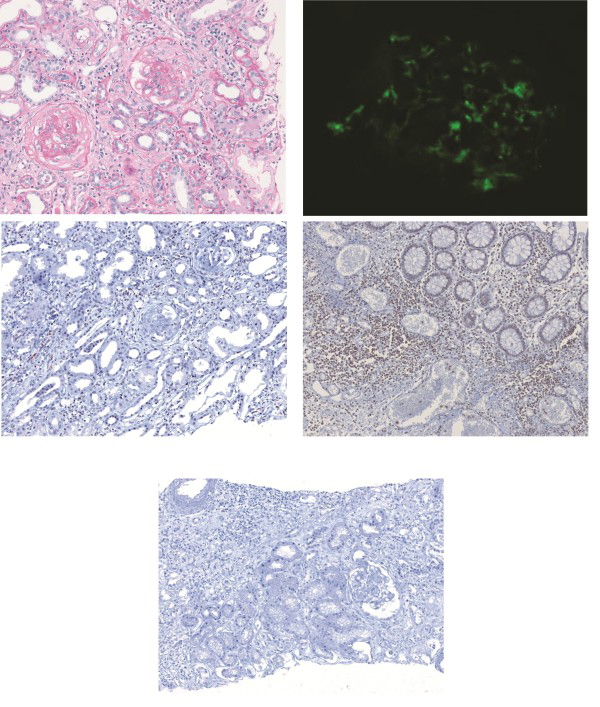
**Histological findings of the renal tissues and intestinal mucosa.** Crescent formations of the glomeruli were observed with periodic acid-Schiff (PAS) staining in light microscopy (**A**: 100× magnification). Immunofluorescence staining was positive for IgA in the mesangial regions (**B**). Immunostaining for interleukin (IL)-17 was positive in renal tissue in April 2011 (**C**) and exacerbated colonic ulcer tissue (**D**) in December 2010. IL-17 staining was negative in the primary IgA nephropathy (control) patient (E: 100× magnification).

**Figure 2:**
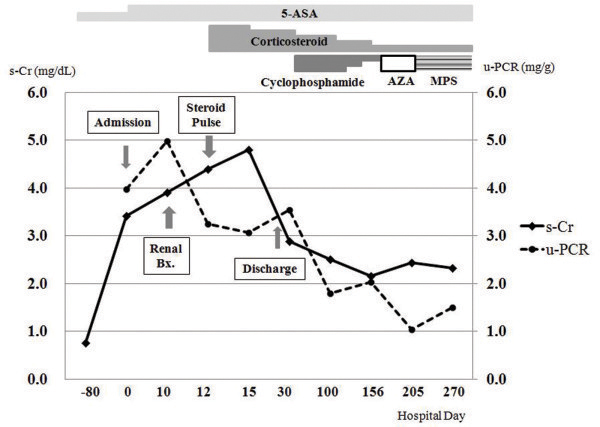
Clinical course and treatment of the patient.

Renal biopsy findings revealed nine crescent formations and eight scleroses in 17 glomeruli. Endocapillary hypercellularity was also present. Inflammatory cells had infiltrated into the interstitium. Mild tubular atrophy with interstitial fibrosis was observed by light microscopy. In the mesangium, immunofluorescence assays revealed 2+ diffuse fine granular staining for IgA and 1+ staining for IgM and C3. Electron microscopy revealed dense deposits in the mesangial area (Figure 1A, B). The patient was subsequently diagnosed with rapidly progressive IgAN. Two days after the renal biopsy was performed, serum creatinine levels increased from 3.9 mg/dL to 4.8 mg/dL. The patient was treated with prednisolone pulse therapy (250 mg twice daily) for 3 consecutive days; the dose was tapered to 1 mg/kg/day (Figure 2). Planned intravenous cyclophosphamide therapy was canceled because the patient developed abdominal symptoms on the fifth day of steroid treatment. He had watery diarrhea 10 times daily, and laboratory data revealed that he suffered from metabolic acidosis caused by severe diarrhea. Blood gas analysis showed the following parameters: pH 7.24, PaCO_2_ 22.4, PaO_2_ 82.6, and HCO_3_ 9.7. His serum chloride level was 121 mmol/L; BUN and serum creatinine levels were 89 and 3.88 mg/dL, respectively. The patient was treated with 5-ASA (2 g/day) and loperamide (6 mg/day). In addition, intravenous fluid therapy was administered to restore volume loss. The watery diarrhea subsided in 2 weeks, and the patient was discharged. He was further treated at the outpatient department with oral doses of deflazacort (48 mg/day) and cyclophosphamide (100 mg/day). In July 2011 (3 months after treatment), these doses were tapered to 24 mg/day and 50 mg/day, respectively. 5-ASA dosage was maintained at 2.0 g/day. The patient’s BUN level was 61.2 mg/dL, and his serum creatinine concentration was 2.5 mg/dL. Proteinuria was 2+ by dipstick test, and the spot urine protein-creatinine ratio was 1.8 mg/g.

In September 2011, cyclophosphamide was replaced with azathioprine (100 mg/day). One month later, the patient was switched from azathioprine to mycophenolate sodium (360 mg/day) because of bone marrow suppression. His intestinal symptoms were not aggravated, and renal functions were also maintained (33.8 mg/dL BUN and 2.32 mg/dL serum creatinine) while taking mycophenolate sodium (360 mg/day) and deflazacort (12 mg/day) during December 2011.

To evaluate pathophysiological characteristics common for the exacerbation of Crohn’s disease and IgAN, as well as possible mediators, we measured interleukin (IL)-17 expression in renal and colon tissues from the patient. We also compared renal IL-17 expression to that in tissues from cases of primary IgAN without Crohn’s disease. Immunostaining for IL-17 was positive in both renal tissue and exacerbated colonic ulcers from the patient (Figure 1C, D), in comparison, this staining was negative in renal tissues from a patient with primary IgAN (control; Figure 1E).

## Conclusions

In this report, we presented a case of rapidly progressive IgAN concurrent with aggravation of Crohn’s disease, both of which responded to immunosuppressive treatment. The association between IgAN and Crohn’s disease is well established and increasingly reported in the literature [4-7]. However, pathophysiological association between the two diseases remains a matter of debate. To our best knowledge, only one case of crescentic IgAN in Crohn’s disease was previously reported, in which acute renal failure occurred 8 weeks after rectosigmoid resection. Unlike the case detailed here, it did not demonstrate a direct association between aggravation of renal function and concurrent intestinal symptoms [3]. IgAN, a type of glomerulonephritis that is common in the general population, especially in Asian populations, might be a coincidental pathology in a patient with Crohn’s disease. However, simultaneous appearance of clinical features of rapidly progressive IgAN and aggravation of Crohn’s disease, as well as our patient’s good response to therapy, suggest a common underlying pathophysiology in both conditions. In addition, in the detailed case, both renal and ulcerated colonic tissue were positive for IL-17, which might further support a potential common immunologic mechanism.

IL-17, a cytokine produced by CD4+ memory T cells, induces the release of pro-inflammatory cytokines from monocytes and macrophages [10,11]. This soluble T cell factor thereby promotes inflammation. Recently, IL-17 was identified as a novel immunoregulatory cytokine involved in Crohn’s disease. Production of this factor is significantly increased in the blood and intestinal mucosa of patients with active Crohn’s disease [12]. Moreover, Lin *et al.* have demonstrated histologically that tubular IL-17 expression is higher in IgAN patients than in normal controls, and IgAN patients with higher IL-17 expression in tubular epithelial cells have reduced renal function and more severe cases of proteinuria [13]. In our patient, both renal tissues and exacerbated colonic ulcers stained positive for IL-17. These first established findings suggest that IL-17 activation may be involved simultaneously in both aggravating intestinal inflammation and promoting the development of rapidly progressive IgAN in patients with Crohn’s disease.

Several cases of IgAN in patients with Crohn’s disease have suggested a possible pathophysiological relationship between the two conditions. Increased intestinal permeability has been observed in patients with both IgAN and Crohn’s disease [14,15]. Altered intestinal permeability may play a role in the pathogenesis of both diseases by promoting the systemic influx of antigens as well as immune complex formation and deposition. Moreover, it has been established that CD4+ lymphocytes are predominant in the peripheral blood of patients with Crohn’s disease [16] and the number of T alpha cells possessing IgA-specific helper activity is increased in patients with IgAN [17]. There is reasonable consensus that abnormal T helper lymphocyte function contributes to the pathogenesis of both diseases. In addition, an association between HLA-DR1 and these diseases has been demonstrated in patients [18,19], which indicates the possible involvement of common genetic factors.

It has been suggested that circulating immune complexes containing IgA play a role in rapidly progressive IgAN, and high levels of circulating immune complexes correlate with the clinical activity and extent of glomerular crescent formation [20]. In this patient, the proteinuria and azotemia intensified as his intestinal symptoms were aggravated. It appears that loss of mucosal antigen exclusion in Crohn’s disease led to increased immune activation, resulting in pathogenic IgA production and the aggravation of IgAN. These possible common mechanisms are supported by a recurrent IgAN case linked to the onset of Crohn’s disease [21]. Moreover, a recent report showing steroid treatment for the bowel disease resulted in both complete remission of Crohn’s disease and improvements in renal function, proteinuria, and hematuria, further supporting the hypothesis of a common pathogenesis [5]. However, whether the clinical activity of IgAN in patients with Crohn’s disease correlates with the progression of gastrointestinal disease remains uncertain. Lee *et al.* encountered a patient who had maintained remission of Crohn’s disease with 5-ASA and azathioprine, but was diagnosed with IgAN [6]. In any case, the possibility that primary unrelated IgAN deteriorates independent of coexistent Crohn’s disease or transitions to a clinically evident disease during a relapse of Crohn’s disease cannot be excluded. Additional studies are needed to determine the causal relationship between the two diseases.

Immunosuppressive agents, including high-dose corticosteroids and cyclophosphamide with or without plasma exchange, are indicated for treating rapidly progressive glomerulonephritis. Regimens similar to those recommended for renal vasculitis can reportedly help preserve renal function [22]. Our patient was treated with steroid pulse therapy followed by high doses of steroids and oral cyclophosphamide. His renal function gradually recovered with this regimen. His intestinal symptoms also improved. Dabadie *et al.* reported a patient with Crohn’s disease showing clinical improvement of gastrointestinal symptoms after receiving treatment for IgAN [23]. Clinical improvement of both IgAN and Crohn’s disease may be expected owing to common pathophysiologic characteristics shared by these diseases. The use of immunomodulatory agents may resolve impaired T cell immunity and production of immune complexes.

Crescentic IgAN has a poorer prognosis than other forms of crescentic glomerulonephritis, even with immunosuppressive therapy. Renal survival rates among crescentic IgAN patients are reportedly only 50% at 1 year and 20% at 5 years [9]. However, cases of rapidly progressive IgAN in Crohn’s disease patients have rarely been published [3]. Further analysis and follow-up of more case series will provide information about the relationship between these diseases and renal survival.

In summary, we experienced a case of rapidly progressive IgAN with exacerbation of Crohn’s disease. The patient responded well to immunosuppressive therapies. Based on a literature review, we suggest that a variety of mechanisms, including intestinal hyperpermeability, abnormal T helper lymphocyte function, and IL-17 activation, might be involved in the common pathogenesis of these two diseases.

## Consent

Written informed consent was obtained from the patient for publication of this case.

## Abbreviations

5-ASA, 5-aminosalicylic acid; AZA, Azathioprine; MPS, Mycophenolate sodium; PCR, Protein-creatinine ratio.

## Competing interests

None of the authors have any competing interests.

## Authors’ contributions

JYC, MKJ, and JHC were the physicians who treated the patient in this report. CHY and HYJ performed the renal biopsy evaluation. YJK performed the pathology studies. The manuscript was prepared by JYC, CDK, YLK, and SHP. All authors participated in discussions about the manuscript and approved the final version.

## Pre-publication history

The pre-publication history for this paper can be accessed here:

http://www.biomedcentral.com/1471-2369/13/84/prepub
